# Social and health system barriers: Investigating Circumstances of Mortality Categories (COMCATs) for deceased patients with T2DM in the sub-national Saudi Arabia register

**DOI:** 10.1371/journal.pone.0313956

**Published:** 2024-11-21

**Authors:** Faleh Alyazidi, Deler Shakely, Fawaz Alyazidi, Lubna A. Alnasser, Max Petzold, Laith Hussain-Alkhateeb

**Affiliations:** 1 School of Public Health and Community Medicine, Institute of Medicine, Sahlgrenska Academy, University of Gothenburg, Gothenburg, Sweden; 2 Department of Public Health, College of Health Sciences at Al-Leith, Umm Al-Qura University, Al-Leith, Kingdom of Saudi Arabia; 3 Infectious Diseases Control Department, Executive Directorate of Preventive Medicine, Makkah Healthcare Cluster, Makkah, Kingdom of Saudi Arabia; 4 Department of Population Health, King Abdullah International Medical Research Centre, King Saud Bin Abdulaziz University for Health Sciences, Riyadh, Kingdom of Saudi Arabia; Ministry of Health, Sri Lanka, SRI LANKA

## Abstract

Health policy debates rely on reliable and timely information on major causes of mortality and their associated attributors, especially to overcome the traditional public health focus restricted to the biomedical cause of death (COD). This study explores relevant social and health system circumstantial barriers to accessing healthcare services among deceased patients with Type 2 Diabetes Mellitus (T2DM) in Saudi Arabia. A total of 302 verbal autopsy (VA) interviews were conducted with relatives or caregivers of the deceased who died between 2018 and 2021, based on T2DM medical records from Alnoor Specialist Hospital in the Western Province, Saudi Arabia. The Bayesian-based InterVA-5 algorithm was employed as a validated source to determine the probable COD and Circumstances Of Mortality Categories (COMCATs) for each case. COMCATs stand for predetermined categories of multiple social and healthcare system circumstances that contribute to an individual’s death. The likelihoods of COD and COMCATs derived from InterVA-5 software were computed independently to generate the ‘cause-specific mortality fractions’ (CSMFs) of the COD and COMCATs. The CSMFs for the seven COMCATs categories were then ranked based on their derived probabilities for the corresponding COMCATs across all major COD categories. The top CODs were circulatory diseases (35.8%), stroke (16.6%), and diabetes mellitus (14.3%). The probabilities of COMCATs indicated that most deaths were attributed to ‘inevitable’ causes (e.g., terminal illness), followed by ‘recognition’ (inability to recognize the severity of illness) and ‘traditions’ (local attitudes deterring patients from seeking medical services on time). Addressing ‘recognition’ and ‘traditions’ barriers could reduce mortality rates and improve access to healthcare, helping the Saudi health system accelerate the progress towards the systematic measurement of key universal health coverage indicators. The study emphasizes the need for a robust and standardized VA method within routine medical services to address factors influencing healthcare access towards improved health outcomes.

## Introduction

Ascertaining the medical cause of death (COD) remains a global challenge [1, 2], with just over half of the world’s mortalities going unrecorded by routine civil registration systems, especially in societies with health inequalities and socioeconomic disadvantages [3, 4]. Reporting and interpreting data on COD and their circumstances is fundamental for identifying avoidable deaths, in order to enable health systems to more effectively react and prevent such deaths at the community level [5, 6]. The lack of data on essential factors such as COD and death circumstances–mainly social and health system factors–contributes to other structural inequalities and adverse health outcomes. A standardized understanding of key circumstantial factors in the pathway to death can illuminate health systems to allocate resources effectively and improve health service accessibility, thereby improving population health. Such systematic assessment can help identify underlying variables, including logistics, barriers to service utilization, and health system responses, which are critical for public health planning and policy development [3].

In settings where medical certification documentation is generally incomplete, the verbal autopsy (VA) method can be a feasible alternative to fill existing gaps [3, 7, 8]. The basic principle of VA involves interviewing the close relatives or final caregivers of the deceased, using a standardized questionnaire, to gather information about the signs, symptoms, and circumstances surrounding the death [4, 9]. The “WHO Verbal Autopsy Instrument” (WHO VA) 2016 collects information about the deceased’s background, details on medical signs and symptoms, injury/accident history, symptom duration, and health services used by the deceased before death [10]. Overall, the WHO VA 2016 questionnaire has 305 items relevant to determining the COD [4].

VA interviews often have complex skip patterns (i.e., when certain questions only apply to specific age or sex groups), making it more efficient to use portable data capture tools like smartphones or tablets to handle these interviews [4, 10]. The VA interview data is later interpreted into probable COD based on the International Classification of Disease (ICD). Although physicians have often performed interpretations of VA data, increasingly automated software such as InterVA-5 are being used to assign causes, which can be consistent, faster, and cheaper [3].

In 2012, ten novel circumstantial items were added to the WHO VA 2012 [11] and retained in the WHO VA 2016, incorporating social and health system dimensions to complement the medical COD profile [3]. These dimensions were referred to as ‘Circumstances Of Mortality Categories’ (COMCATs), which are primarily concerned with ranking social, economic, and logistical healthcare seeking and utilization at and around the time of death [4]. Furthermore, the ten questions related to COMCATs are published elsewhere [7]. COMCATs are classified into seven categories: ‘traditions, emergencies, recognition, resources, health systems, inevitability, and multiple’ [3, 7], as described in Table 1.

**Table 1:** Circumstances Of Mortality CATegories (COMCATs) [7].

COMCAT	Definition
**Traditions**	Traditional practices or beliefs influenced health-seeking behavior and the pathway to death
**Emergencies**	Sudden, urgent or unexpected conditions leading to death, which probably precluded life-saving actions
**Recognition**	Lack of recognition or awareness of serious disease (e.g., symptoms or severity) negatively influenced health-seeking behavior
**Resources**	Inability to mobilize and use resources (e.g., material, transport, financial) hindered access to health care
**Health Systems**	Problems in getting health care despite accessing health facilities (e.g., related to admissions, treatments and medications)
**Inevitability**	Death occurred in circumstances that could not reasonably have been averted (e.g., very elderly or recognized terminal conditions)
**Multiple**	A combination of the above categories affected the pathway to death; no single factor predominated

As health outcomes and responses are not normally distributed worldwide, ways to routinely assess circumstantial factors contributing to deaths can offer valuable insight [7]. A framework that places medical outcomes within cultural, social, and health system contexts can better inform health planning for preventable deaths [7]. This parallel approach does not seek to replace the traditional medical cause classifications, rather it adds another perspective to understanding death, permitting large-scale assessment and classification of mortality circumstances at the population level [3]. With the current emphasis from the WHO on achieving Universal Health Coverage, standardized tools that can effectively link health service utilization to mortality outcomes in populations will be increasingly necessary for public health strategies.

One of the fundamental aims of the Sustainable Development Goals (SDGs) is to ensure equitable and unbiased access to basic primary health services for people worldwide, and to ensure that measures are in place to make their utilization possible [12]. The Kingdom of Saudi Arabia’s ‘Department of Health Affairs’ aims to provide comprehensive health care for all in an equitable and accessible manner [13]. Addressing the barriers to accessing healthcare services among the geographically and socially diverse Saudi population will be essential in the undergoing health system re-engineering towards the Saudi 2030 Vision. Vision 2030’s ‘Health Sector Transformation Program’ seeks to transform the health sector into a comprehensive, efficient, and integrated health system that prioritizes the health of individuals and society, including citizens, residents, and visitors [14]. The latter is a particularly important consideration in Saudi Arabia, as it receives millions of pilgrims annually for Hajj and Umrah; the national health infrastructure, particularly in the western region, has a significant responsibility of ensuring universal and equitable healthcare access for a vast influx of people who come to perform Hajj and Umrah from all over the world [13, 15, 16].

Data from several studies has explored barriers that hinder healthcare accessibility in different regions in Saudi Arabia [17–19]. A Saudi study investigating factors influencing access to and use of primary care centers in urban and rural areas revealed important differences between urban and rural populations [17]. It identified that the distance to the primary health center was one factor that hindered access to healthcare services among rural patients, which is typically considered to be a health system threat. However, the study lacked a comprehensive approach, particularly it did not consider social and health system factors that influence healthcare services-seeking behavior, which would have given insights into the required service provision [17].

Another study conducted in Hail City, found that the level of satisfaction among primary healthcare users was attributed to health system factors, specifically difficulties in accessing medical care and the availability of doctors [18]. Furthermore, a study conducted in Makkah city, which hosts the Muslim pilgrims, aimed to determine the variations in access to public hospitals using maps and geographical information systems. It revealed that the majority of health facilities were located in urban areas [19], which may influence and delay prompt access to essential healthcare services. However, the study findings were limited in terms of the relatively small sample, relying on population data from a decade ago, and the use of geographical information systems, which can be unreliable to understand specific health-seeking behaviors. The study left open questions about the potential effects of dynamic changes in population on access to healthcare.

Nevertheless, it would be crucial to revise and determine key social and health system aspects preventing individuals from seeking medical services, using standardized methods. For instance, there is a need to explore how local beliefs and behaviors [20], the ability of people to recognize severe illnesses, and how logistical resources delay people from accessing healthcare services, among avoidable factors. Ideally, using a systematic instrument to explore these factors influencing healthcare-seeking behaviors among the Saudi population will enable comparisons across time and space, which has hitherto been lacking in previous research.

There is a particularly high prevalence rate of diabetes in Saudi Arabia [21–23], and diabetes represents a substantial public health concern worldwide, increasing strain on the healthcare system due to diabetes itself, and various associated complications and co-morbidities [24]. Effective diabetes management requires many follow-up visits and regular medical service appointments. Nonetheless, concerns persist regarding social and health system factors that can hinder access to healthcare services, which is a critical issue in the current global and national public health agenda. Despite the interest in research and health policy in Saudi Arabia, there is a lack of evidence of non-medical factors, such as social and health system barriers, that may have contributed to deaths in addition to medical causes, which can be explored by novel methods such as VA.

To address the identified gaps, this study aims to identify and scrutinize circumstantial factors related to deaths, including social and health system barriers to accessing healthcare services. The study further assesses the sensibility of COMCATs in a Saudi context and examines the demographic determinants and time factors associated with these barriers among deceased patients with T2DM in Saudi Arabia, using the standardized WHO VA 2016 tool.

## Methods

### Study design and population

A sub-national register-based cross-sectional study was conducted in Makkah City, Saudi Arabia. We obtained data from 1,609 deaths recorded in the T2DM medical register at Alnoor Specialist Hospital between 2018 and 2021. The study site, is a key public healthcare facility in Makkah City, serving a population of 2.5 million, and significantly contributing to healthcare services in the region.

A sample of 311 deceased patients was predetermined based on finite sampling for a proportion assumed to be 50%, with a significance level of 5% [25]. Since the VA concept is relatively novel in the Saudi culture, especially among the general public, and the nature of recollecting traumatic and fatal experiences is typically associated with emotional distress, a fair response rate was expected, which was assumed at about 50%. Therefore, to meet the pre-estimated 311 sample required for this study assessment, 700 cases were selected using simple random sampling from the overall 1609 deaths of the T2DM register, utilizing the “Rand” function in Excel. Relatives or close caregivers of these cases were then contacted and consented to conduct the VA interview. Ultimately, 302 VA interviews were completed and retained for data processing after excluding cases of refusal to participate, and incorrect or unreachable contact information.

### Data collection

The characteristics and background of the deceased were gathered from the medical records at Alnoor Specialist Hospital. Death records captured vital information such as the date and place of death, sex, age, and contact information of their next of kin, along with ICD-10 codes for the COD, with additional physicians’ remarks describing the circumstances of COD. Other socio-demographic factors were also considered, such as nationality, marital status, and education, in order to account for the population heterogeneity expected in the dynamic population of this province (given the presence of pilgrims from around the world).

COVID-19 restrictions posed a challenge to conducting real-life face-to-face VA interviews, especially in Saudi Arabia, where strict physical distancing preventive measures were applied to prevent COVID-19 transmission [26, 27]. Therefore, telephone interviews were used as alternatives to face-to-face interviews. VA interviews were conducted with the families of the deceased using the WHO VA 2016 questionnaire, downloaded on the Open Data Kit (ODK) on Android smartphones. The VA interviews were conducted by the first author and a trained research assistant, with each VA interview lasting an average of 21 minutes.

The interviews were conducted in Arabic without audio recording, but VA data inputs were reviewed and safely stored at the university’s electronic databases after removing all personal identifiers. We followed the WHO’s VA Field Interviewers Manual [28] that VA interviewers should have a good working knowledge of the relevant local language and the language used in the VA questionnaire. Another criterion is that interviewers should be aware of the cultural context and trusted and accepted by the community. The VA interviewers in this study met these criteria, and additionally underwent comprehensive training before the commencement of the VA data collection. The training included detailed sessions with VA experts, and online workshops were held on the objectives and importance of VA interviews, ethical considerations, and step-by-step instructions on how to conduct the interviews using the standardized WHO VA 2016 questionnaire.

### Data management

The deceased’s age was categorized into four age groups (34–59, 60–69, 70–79, and 80+ years old), and the levels of education were grouped as ‘*illiterate*’, ‘*basic education (including primary*, *secondary school*, *and high school)*’, *and* ‘*advanced education*, *including university level*’. The relationship between the VA informants and the deceased was classified into two groups: (‘first-degree’ and ‘second-degree relative’), see (S1 Table), whereby the deceased’s marital status was dichotomized as single and married. The place of death was recorded and processed as binary information (i.e., ‘home’ or ‘hospital/health facility’). The VA COD were further categorized into broader categories, as shown in (S2 Table).

### Interpretation of VA data

After conducting VA interviews using WHO VA 2016 questionnaire, each VA interview data was processed and analyzed using InterVA-5 software, which assigns up to three likely COD and COMCATs with associated likelihoods. InterVA-5 is one of the most widely used software solutions that uses a probabilistic algorithm to interpret VA data, including COMCATs information [4]. The software is open-source, accessible, and compatible with WHO’s VA standards. InterVA-5 has been validated for VA and COMCAT in other studies [3, 7]. During the development of the open-source InterVA-5 model [4], a Bayesian probabilistic sub-model was created to transform the indicators obtained from each VA case into likelihoods for the six COMCATs. The statistical methodology and conceptual basis of InterVA-5 version were identical to those of previous InterVA models as described in earlier research [3, 29].

The medical COD model links signs and symptoms to a particular COD by analyzing a database of conditional prior probabilities, while the COMCAT, which is a sub-model of InterVA-5 considers all the indicators gathered during the interview, and processes conditional probabilities that link each response to a specific COMCAT category [3]. During this process, InterVA-5 software assigns each individual to one of the seven COMCATs categories. If the calculated likelihood from the WHO VA 2016 items exceeds 50% for one of the six COMCATs, it will be assigned to the individual to determine the terminal COMCATs category. Otherwise, the individual would fall under the seventh ‘multiple’ COMCAT category when the calculated likelihood does not reach 50% or exceeds 50% for more than one COMCAT category [3, 7].

### Data analysis

The likelihoods of COD and COMCATs derived from InterVA-5 software were computed independently to generate the causes-specific mortality fraction (CSMF), which estimates the proportion of each COD category to the total number of deaths [30, 31]. The CSMF of the seven COMCATs categories were then ranked based on their derived probabilities for the corresponding COMCATs across all major COD categories, which range between 0 and 100% (i.e., rank 1 is assigned to the highest percentage, and rank 7 is assigned to the lowest). Additionally, the distribution of CSMF for each COD and their corresponding COMCATs category was stratified by time, age groups, and sex. Numerical and graphical descriptive statistics were utilized to summarize the trend and pattern of the VA data outputs. STATA Release 17.0 software was used for all statistical analyses.

### Ethical considerations

This study received ethical approval to conduct VA interviews from the Institutional Review Board (IRB) at Makkah Health Affairs, Ministry of Health, Saudi Arabia (IRB Number H-02-K-076-0321-478). The study adhered to the principles outlined in the Declaration of Helsinki. While contact details of the deceased’s families were initially provided for VA interviews, any identifying information regarding the deceased and their families was removed from the data to ensure confidentiality and anonymity post-interview. Subsequently, a newly encrypted dataset with anonymized IDs replaced the original data for analysis and storage. The informed consent process was formally documented through the utilization of the IRB-approved consent form.

Due to stringent COVID-19 safety measures and to minimize potential emotional distress, the VA process was conducted remotely through telephone calls over four months, from 1 April 2021 to the end of July 2021. At the commencement of the VA interview, the interviewers demonstrated the content of the consent form, with a clear statement about the participant’s option to either decline or proceed with the interview. Upon declaration by the respondents, the VA interviewers documented the consent’s date, time, and status as approved (if the respondent agreed and continued to the end of the interview) or rejected (if the respondent declined to participate or chose not to continue after initial agreement). All rejected interviews were excluded from the dataset and subsequent analysis. As this consent was verbal in nature, the VA interview assistant (FA) witnessed this consent process.

As part of the VA training, ethical considerations and sensitivity training were considered to handle bereaved families with care and respect. Beyond the fully informed consent at the start of each interview, the VA interviewers were trained to provide emotional support to the relatives/families throughout the VA interview process. Participants were contacted to schedule the interviews at their convenience. During the interviews, participants were informed that they could pause or stop the interview at any time if they felt uncomfortable. Furthermore, interviewers were instructed to be attentive to participants’ emotional cues and to offer to pause or change the topic if needed, as well as to ensure that the emotional well-being of the participants was prioritized at all times.

## Results

### Summary of overall background and characteristics of study participants

During the period 2018–2021 period, 1609 deaths were identified at Alnoor Specialist Hospital T2DM register. From these cases, 302 deceased relatives or caregivers were contacted and consented to conduct the VA interview over four months, with only three of the respondents being females. InterVA-5 assigned either a single, two, or three likely COD along with COMCATs attributed to deaths. Table 2 presents the background and characteristics of the 302 death cases.

**Table 2:** Background and characteristics for 302 death cases in Makkah region, Saudi Arabia.

Background characteristics	Distribution of deaths *n* (%)
**Age group**	
34–59	68 (22.5)
60–69	88 (29.2)
70–79	84 (27.8)
≥80 years	62 (20.5)
**Sex**	
Female	126 (41.7)
Male	176 (58.3)
**Respondent’s** (interviewee) relationship to the deceased	
First degree relationship	260 (86.1)
Second degree relationship	42 (13.9)
**Education level of the deceased**	
Illiterate	121 (40)
Basic education	137 (45.4)
Advanced education	44 (14.6)
**Year of death**	
2018	28 (9.3)
2019	69 (22.8)
2020	161 (53.3)
2021	44 (14.6)
**Marital status**	
Single	116 (38.4)
Married	186 (61.6)
**Place of death**	
Home	60 (19.9)
Hospital	242 (80.1)

### Cause-specific mortality fractions and corresponding COMCATs, and their ranks

**Fig 1:**
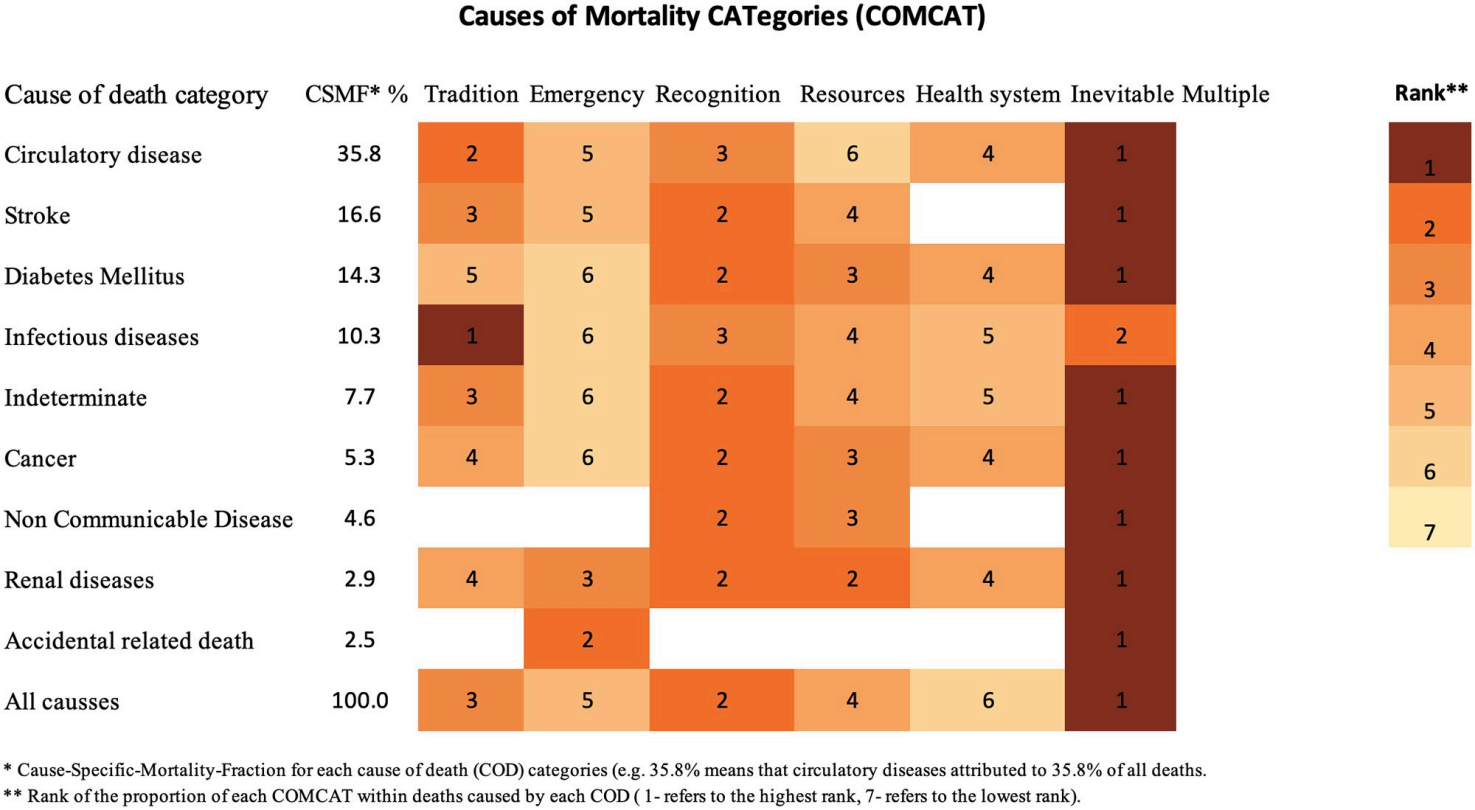
Cause-specific mortality fractions (CSMF) and corresponding Circumstances of Mortality Categories (COMCATs) and their ranks for each major cause category for 302 deaths in Makkah City, Saudi Arabia.

(Fig 1) displays the CSMFs (expressed in percentages), for each cause group, with ranked corresponding COMCATs attributed to deaths. In descending order, the four leading COD were circulatory-related deaths (35.8%), stroke (16.6%), diabetes mellitus (DM) (14.3%), and infectious diseases-related deaths (10.3%) (including HIV/AIDS, pulmonary tuberculosis, acute pneumonia, and other unspecified infectious diseases). According to the InterVA-5 algorithm, about 8% of all deaths were classified as indeterminate causes, while 5.3% were attributed to cancer, 4.6% to NCDs, 2.9% to renal diseases, and 2.5% to accident-related deaths.

According to the analysis of COMCATs in InterVA-5, ‘inevitability’ was the top-ranked category, followed by ‘recognition’, and adherence to local ‘traditions’ categories. The InterVA-5 did not assign the seventh category (‘multiple’ COMCATs) to any case, which indicates the highly distinguished categories observed in this data. For deaths related to circulatory diseases, which are the most prevalent COD, traditional beliefs, ‘recognition’, and issues related to the ‘health system’ were identified as top circumstances contributing to these NCDs deaths, while ‘resources’ were the least cited.

Deaths were attributed with COD issues related to: ‘recognition’, ‘traditional beliefs’, and ‘resources’ for stroke and indeterminate causes; ‘recognition’, ‘resources’, ‘health system’, and ‘traditions’ for DM and cancer. ‘Emergencies’ COMCAT was ranked the last, but accident-related deaths were highly linked to this category as the primary factor contributing to such deaths. Infectious disease-related deaths were primarily attributed to ‘traditions’ followed by ‘recognition’, ‘resources’, and the ‘health system’ ranking, while ‘emergencies’ again had the lowest prevalence. ‘Recognition’ and ‘resources’ were identified as the main circumstances contributing to NCD-related deaths. The main circumstances contributing to renal diseases-related deaths were ‘recognition’, ‘resources’, ‘emergencies’, and ‘traditions’, with the ‘health system’ having the least attribution.

### The distribution of CSMFs of COD and their corresponding COMCATs, stratified by age groups

**Fig 2:**
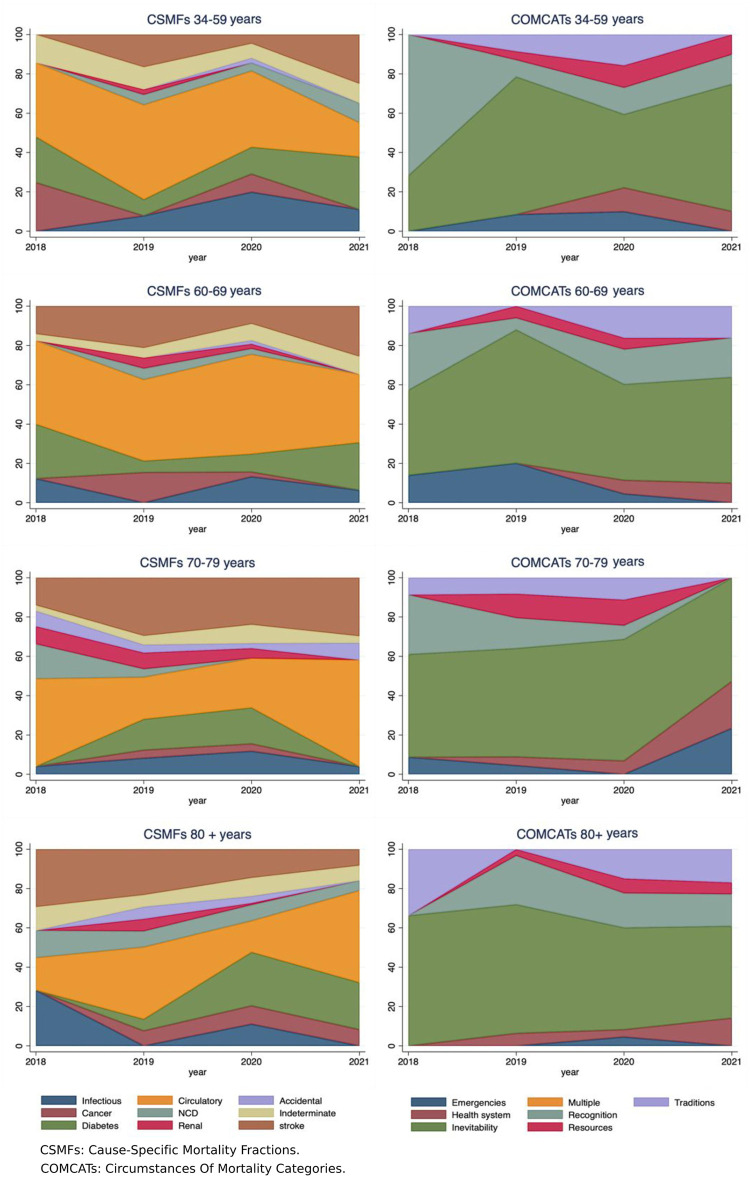
The distribution of CSMF and their corresponding COMCATs among 302 deaths between 2018–2021, stratified by age groups, in Makkah City, Saudi Arabia.

(Fig 2) illustrates the CSMFs of COD and COMCATs by age group and the year of death. VA data revealed distinct patterns regarding the leading COD and circumstantial categories when stratified by age groups. Circulatory diseases, stroke, and DM were found to be the most common medical CODs across all age groups. Correspondingly, the circumstantial COD was predominantly attributed to ‘inevitability’ as the top-ranked category across all age groups.

It is worth noting that stroke deaths were less common among deceased patients aged 34–59 compared to older age groups, while infectious diseases were more prevalent among those in this age range. This showed an increasing trend from 2018, which decreased slightly in 2020. Most deaths in the 34–59 age group were attributed to the ‘recognition’, ‘resources’, and ‘traditions’ circumstances as avoidable barriers.

Furthermore, among the 70–79 age group, a significant proportion of mortality was attributed to circulatory diseases and stroke, with a substantial increase observed in ‘health system’ and ‘emergencies’ categories, particularly from 2020 to 2021.

Circulatory diseases were the leading COD among deceased aged 80 and above, followed by DM and stroke. Notably, the deaths in this age group were attributed to ‘recognition’ and ‘traditions’ COMCATs categories, while constraints arising from ‘emergencies’ were not found frequently in this age group, despite the presence of accident-related CODs.

### The distribution of CSMFs of COD and their corresponding COMCATs, stratified by sex

**Fig 3:**
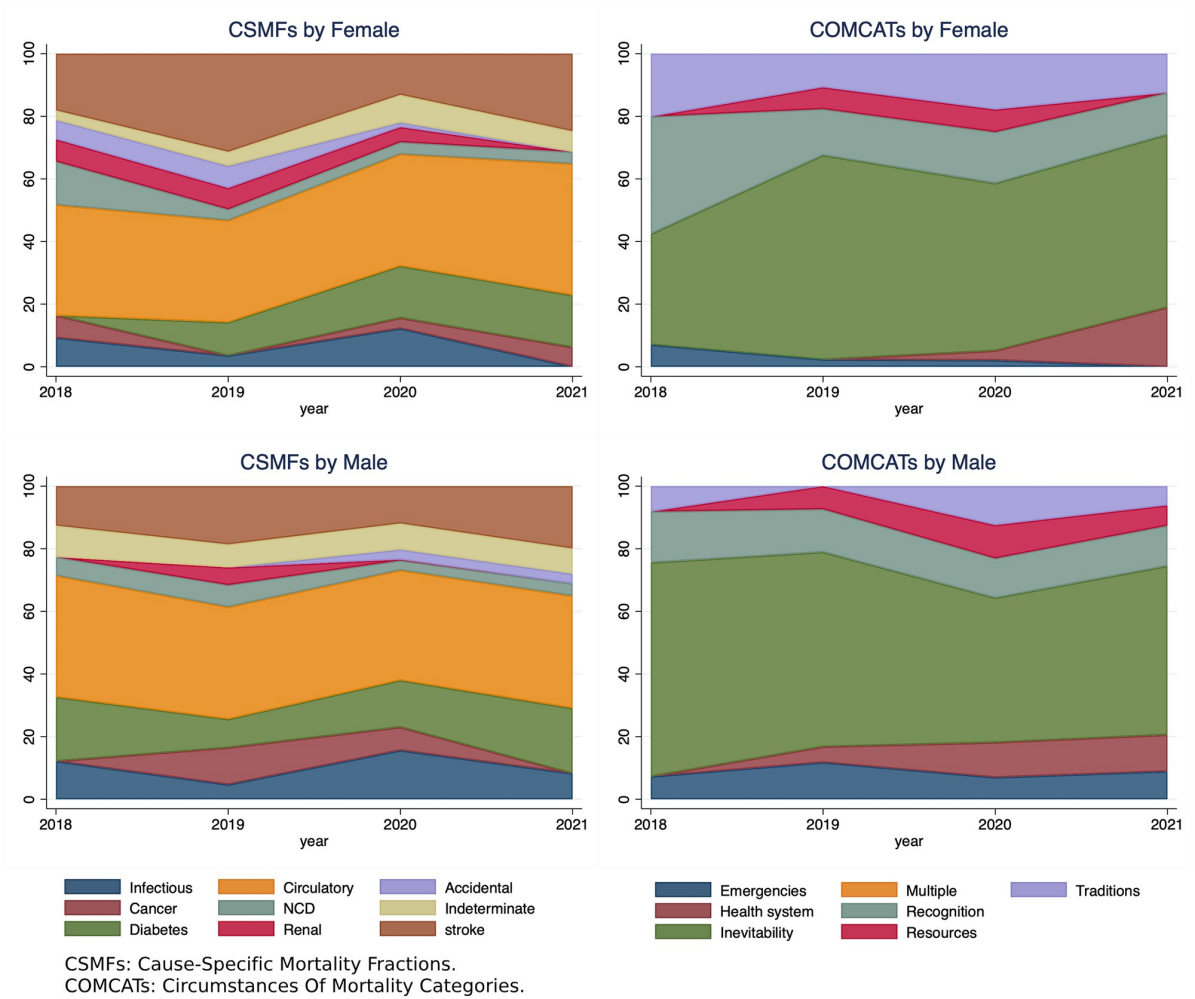
The distribution of CSMF and their corresponding COMCAT of 302 deaths between 2018–2021 stratified by sex, in Makkah City, Saudi Arabia.

(Fig 3) displays the distribution of CSMFs of COD and COMCATs by sex over the years. When categorized by circumstantial categories, the majority of deaths for both males and females were classified as ‘Inevitable’, followed by issues related to ‘recognition’ and ‘traditions’. The figure also reveals that ‘traditions’ and ‘health system’ COMCAT categories were more frequently observed in females, with an increasing trend noted from 2020 to 2021. Conversely, constraints related to ‘recognition’ and ‘emergencies’ showed a decreasing trend over time; both ‘emergencies’ and ‘resources’ COMCATs categories were found to be more common in males.

## Discussions

### Putting key findings in Saudi context

Given the global and national population health agenda, this study aims to identify circumstantial factors associated with COD, such as social and health system barriers to accessing healthcare services among deceased patients, using the T2DM sub-national register in Saudi Arabia. Within the Saudi health system, significant reforms are underway to align with the national development blueprint, ‘*Vision 2030*’ [13], and achieve the SDGs, including SDG 3.8 (‘achieving universal health coverage’). Throughout the data collection period, COVID-19 was a substantial challenge to already struggling vital data registries, with unequal access and opportunities for treatment due to the overstretched health systems in many countries, including Saudi Arabia. This triggered the need for counting and accounting for people’s adequate access to timely healthcare services, particularly during public health emergencies like the COVID-19 pandemic [32].

VA has a comprehensive scope that extends beyond the limited medical COD to the causes of mortalities categories incorporated within COD, including logistical, social, and health system causes associated with deaths [3]. The COMCATs model was integrated into VA to enable a comprehensive assessment of disease burden, considering the needs and behaviors of individuals and the responsiveness of the healthcare system in addressing these needs [3, 7]. To the best of our knowledge, the COMCATs concept used in this study is an entirely novel approach in Saudi Arabia to extract an additional dimension of COD information. The rationale for incorporating mortality patterns and trends, including COD and other relevant factors (such as social and health system factors), into the Saudi healthcare system is that this can provide crucial information for public health policy, and enable more effective planning of healthcare services to enhance the overall health of the population.

For example, there is a major difference in circumstances between an individual with DM in a remote rural area, who has little choice but to remain at home to manage his treatment protocol, and a DM patient closer to a health facility, who can easily avail himself of a facility consultation and follow-up care. Both individuals may die of DM-associated disease, but adding COMCATs to the VA process could circumstantially differentiate the two cases of resource and health systems limitations. Thus, the COMCATs system adds insights to VA findings that are relevant to local health systems monitoring, access to healthcare, and resource allocation but with minimal additional effort and cost.

DM is a chronic condition that requires ongoing healthcare management, including access to healthcare services and recognition of symptoms [33, 34]. This study conducted VA interviews with the final caregivers or relatives of the deceased patients with T2DM. In the context of other CODs, such as circulatory diseases, stroke, infectious diseases, and renal diseases, DM can be a significant risk factor. DM increases a person’s risk of developing heart disease and stroke, which can lead to fatal outcomes [35]. Furthermore, DM can impair the immune system, making people more vulnerable to infectious diseases such as pneumonia [36]. Renal diseases, such as diabetic nephropathy or renal failure, can develop due to poorly managed DM and progress to end-stage renal disease, a severe and life-threatening condition [37]. DM, with a prevalence of 17.7% among Saudi adults [23], is a substantial health concern, imposing a significant burden on the Saudi healthcare system. Accordingly, it is imperative for public health policies and interventions to prioritize addressing the barriers to healthcare access and promoting the early recognition of DM symptoms and complications. This is critical in managing this chronic condition and reducing the risk and burden of DM-related deaths.

In line with the leading CODs in Saudi Arabia [38–42], the findings from this study show that circulatory diseases, stroke, and DM collectively constituted the majority (66.7%) of all deaths. Furthermore, given the high prevalence of chronic diseases such as cancer, diabetes, renal diseases, and other NCDs in Saudi Arabia [21, 23, 24, 43–47], deaths related to these conditions are likely to be classified primarily as ‘inevitable’ within the COMCATs system (i.e., death occurred in circumstances that could not reasonably have been averted). Notably, a large proportion of deaths in this study occurred in hospitals (80%), which could contribute to an increase in the ‘inevitable’ COMCAT category. This may be attributed to some of these deaths resulting from patients being in the final stages of their illness, such as terminal cancer, or being too old to receive effective treatment, as well as instances where patients were brought to hospitals in critical conditions, leading to an inevitable outcome.

Nevertheless, it is worth mentioning that the ‘health system’ COMCAT category, which is related to problems in getting health care despite accessing health facilities (e.g., admissions, treatments, and medications), may not be a significant factor contributing to these deaths, as the majority of deaths occurred in hospitals, suggesting that the deceased had access to health facilities and likely received satisfactory medical care. Furthermore, the study findings that a lack of recognition of the severity of these diseases was an apparent constraint aligns with this context. A lack of recognition or understanding of the severity of these diseases may lead to delayed healthcare-seeking and poor disease management, resulting in negative health outcomes. Therefore, the classification of circumstances as primarily ‘inevitable’ in the COMCATs system, along with the findings of a ‘lack of recognition’ as a significant constraint, provide evidence that these diseases are complex and require a comprehensive approach to prevent and manage these diseases. However, implementing effective interventions to address the identified social and cultural factors may be challenging in Makkah City, as it is a diverse region with multiple cultural and social norms and serves as the host of the annual Hajj and Umrah. Thus, a tailored approach considering these factors is essential to effectively managing chronic conditions and preventing undesirable outcomes.

The rankings as shown in (Fig 1) demonstrate that COMCATs patterns differ between some major groups of medical COD. This is not surprising, and it confirms the reliability and plausibility of the COMCATs model [3, 7], as it consistently aligns medical COD with their associated COMCATs, as demonstrated by several examples. For instance, although accidents accounted for a small proportion of deaths in this study, it is highly likely that deaths caused by accidents are predominantly linked to the ‘inevitability’ and ‘emergency’ COMCATs, mainly if the accidents resulted in an immediate fatality.

Furthermore, the study found that infectious disease deaths were associated with ‘traditions’ and ‘recognition’ as leading COMCATs. It could be speculated that people with infectious diseases tend to rely more on traditional practices rather than seeking healthcare services, due to familiarity with traditional herbal remedies as well as social stigma in some cases. Stigma can play a major role in how some communities perceive and address infectious diseases [48, 49]. This pattern is supported by similar findings in Saudi Arabia [49, 50] and elsewhere, which reported a statistically significant relationship between some infectious diseases and stigma [48]. Therefore, access to healthcare services can be delayed among individuals with infectious diseases, resulting in worse health outcomes.

The study’s findings also highlighted another factor that may significantly shape circumstances contributing to deaths among infectious disease cases: ‘lack of recognition’ of the severity of illness. This may lead to delays in seeking timely medical attention and ultimately result in adverse health outcomes. Another point to consider is that the COVID-19 pandemic affected various aspects of everyday life, and the impacts of lockdowns on healthcare systems continue to reduce access to healthcare [51, 52], which could have influenced the patterns of COMCATs associated with different COD, extending beyond infectious diseases. Efforts to address infectious diseases must consider the social and cultural factors influencing how these diseases are perceived, treated, and managed in different communities, relative to the expedient availability of healthcare resources.

Cause-specific mortality statistics by age and sex are fundamental health data for any country [53]. Based on the VA data, circulatory diseases, stroke, and DM were common medical COD across all age and sex groups. Similarly, ‘recognition’ and ‘traditions’ were the primary circumstantial COD, reflecting avoidable barriers to seeking healthcare. The prevalence of stroke as a major cause of illness and death in Saudi Arabia [45, 54] and elsewhere [55] has been growing rapidly, with age serving as a risk factor for stroke [54, 56]. Notably, our study found that deaths attributed to stroke were less common in the 34–59 age group compared to other older age groups. This aligns with existing literature, as the risk of stroke increases with age [54–56], and is exacerbated by the coexistence of comorbidities such as DM and hypertension in older populations [55].

Our findings showed that deaths attributed to infectious diseases were more common in the younger age group (34–59) compared to other older age groups. This displayed an upward trend in 2018, followed by a marginal decline in 2021. This may be because younger people typically have increased susceptibility to infectious diseases due to adopting more active lifestyles and traveling. The leading COMCATs categories of deaths in the 34–59 age group were primarily ‘inevitable’, followed by ‘recognition’ COMCATs category. When individuals are unaware of the illness severity and associated symptoms, their health-seeking behavior can be adversely affected, and this may explain the increase in the ‘inevitability’ category in this age group.

In the 70–79 age group, COMCATs categories had an upward trend in 2020 of issues concerning accessing healthcare services and emergencies, likely due to the higher proportion of deaths related to circulatory diseases and stroke in this group. Risk factors such as hypertension and diabetes can increase the likelihood of these conditions [57], but inadequate and timely healthcare services can also contribute to poor outcomes. In Saudi Arabia, improving digital health tools utilization, like ‘Sehhaty’, can enhance healthcare access and quality, aligning with Vision 2030 goals. This may improve patient engagement and self-management, communication between patients and healthcare providers, and ultimately health outcomes.

The study indicated overall similarities in COD/COMCAT categories when stratified by sex. However, there were some avoidable sex disparities across certain COMCAT categories. In particular, the ‘traditions’ and ‘health system’ categories were more frequently identified as substantial barriers influencing health-seeking behaviors and the pathway to death among females, with an increasing trend in the ‘health system’ COMCATs category noted from 2020 to 2021. In many cultures, social norms, personal preferences, or modesty concerns may dictate that females prefer to be treated by female healthcare providers [58]. This could partially explain the increased ‘traditions’ and ‘health system’ COMCATs acted as barriers to accessing healthcare among females in this study.

In contrast, males were more prone to issues related to ‘resources’ and ‘emergencies’ COMCATs categories. This could be due to the higher proportion of chronic conditions-related deaths such as cancer, diabetes, and circulatory diseases among males compared to females in this study, given that most accident-related deaths were among the male group. These chronic conditions require ongoing medical care, medications, and frequent visits to healthcare providers, which can be costly and a strain on the family’s budget [3]. Additionally, the ‘resources’ COMCAT category (i.e., inability to mobilize and use resources, such as transportation, material, and financial resources) may have been exacerbated by COVID-19 national preventive measures aimed at reducing the infection transmission, potentially leading to limited or hindered access to healthcare services for males. Therefore, addressing these challenges necessitates a comprehensive approach considering the various healthcare access and utilization barriers.

### Sensible applicability

The practical utility of the VA and COMCATs model lies in their ability to inform public health policy and resource allocation. VA and COMCATs provide cost-effective ways to estimate medical COD and associated factors in settings with incomplete vital registration. The COMCATs model was developed for health service managers and planners seeking standardized methods to modify the pathway-to-survival in a society [3]. The COMCATs model assigns circumstantial categories associated with deaths and classifies information on social and health system factors. Although cause-specific mortality has traditionally been a fundamental analytical tool in health policy, various fundamental drivers and influences contributing to mortality may be concealed within conventional mortality statistics.

In Saudi Arabia, the COMCATs model has public health implications, as it can identify specific areas for intervention to reduce mortality rates and improve access to healthcare, particularly among the most disadvantaged groups in society who may have limited access to healthcare resources. The findings from this study suggest that using standardized categories in COMCATs makes it easier to compare mortality data across different age groups (Fig 2), time periods, and sex (Fig 3), facilitating progress toward global health targets like the SDGs as well as national socioeconomic development (i.e., Vision 2030’s targets). Collectively, combined data from VA–COMCATs can help health policymakers and planners identify trends and patterns in causes of mortality, prioritize public health concerns, and inform the development of targeted interventions.

### Strengths and limitations

VA is an essential tool for determining COD, particularly in areas with limited vital registration systems. However, certain limitations must be considered when interpreting our study findings. Although the small sample size in this study may limit the generalizability of the results, it is worth noting that the sample represents the register data obtained from a major specialist hospital in Makkah city, where most provincial cases are reported. Furthermore, despite employing structured interviews, a standardized WHO VA questionnaire, and relying primarily on first-degree respondents, the potential for recall bias in the VA process remains a limitation that cannot be fully eliminated.

Furthermore, one potential issue with VA interviews is the risk of selection bias. This can be due to the dependency of subjects with correct contact details and reluctance to participate (i.e., refusing to participate or withdraw from the interview). In addition, due to COVID-19 physical distancing measures during the data collection period, VA telephone interviews were conducted instead of face-to-face interviews. However, several studies have shown that telephone interviews can be as effective as face-to-face interviews [59, 60]. One study reported that the results of VA telephone interviews were consistent with previous literature, demonstrating that this method is feasible, accepted by caregivers and healthcare workers, and produces reliable data quality [61]. Despite these limitations, conducting distant VA interviews on a small sample in an area where VA is not commonly used can be considered a strength from an ethical standpoint. This approach has the potential to reduce unwanted emotional or cultural consequences.

## Conclusion

This study applied a standardized VA method to determine the circumstantial categories related to COD, including social and health system barriers to accessing healthcare services among deceased patients with T2DM in Makkah Province. Circulatory diseases, stroke, and DM were found to be the most common medical COD across all age and sex groups. Although ‘inevitable’ COD (terminal illness) dominated the COMCAT categories across almost all CODs, it is crucial to note that barriers related to ‘recognition’ and ‘traditions’ COMCAT categories were found to be avoidable obstacles that hindered healthcare-seeking behaviors. The study showed the sensible value of the COMCATs system in informing health-related decisions, expanding information on avoidable deaths, and monitoring progress toward universal health coverage and the national agenda. The system can potentially monitor disadvantaged groups, ensuring they receive more responsive care and promoting health equality. The study recommends utilizing this tool and translating the findings into effective public health policies to address the barriers that affect healthcare access and enhance health outcomes.

## Supporting information

S1 Table Respondents’ relationship to the deceased. (PDF)

S2 Table Categorizing of causes of death reported by verbal autopsy. (PDF)
