# Supplementary material for: Social and health system barriers: Investigating Circumstances of Mortality Categories (COMCATs) for deceased patients with T2DM in the sub-national Saudi Arabia register
Source: PLoS One. 2024 Nov 21;19(11):e0313956. doi: 10.1371/journal.pone.0313956 (PMC11581326; doi:10.1371/journal.pone.0313956)
Supplement: S1 Table — (PDF) [file pone.0313956.s001.pdf]

S1 Table: Respondents' relationship to the deceased

**First degree relative**	**Second degree relative**
Son	Nephew
Brother	Uncle
Wife	Cousin
Husband	Friend
Sister	Employer
Daughter	
Grandson	
Stepson	
Son in law	
