# Supplementary material for: Social and health system barriers: Investigating Circumstances of Mortality Categories (COMCATs) for deceased patients with T2DM in the sub-national Saudi Arabia register
Source: PLoS One. 2024 Nov 21;19(11):e0313956. doi: 10.1371/journal.pone.0313956 (PMC11581326; doi:10.1371/journal.pone.0313956)
Supplement: S2 Table — (PDF) [file pone.0313956.s002.pdf]

S2 Table: Categorizing of causes of death reported by VA

**Category**	**Cause of death**
Infectious diseases	Sepsis (non-obstetric)<br>Acute resp infect incl pneumonia<br>HIV/AIDS related death<br>Pulmonary tuberculosis<br>Other and unspecified infect diseases
Cancer	Digestive neoplasms<br>Respiratory neoplasms<br>Breast neoplasms<br>Other and unspecified neoplasms
Diabetes mellitus	Diabetes mellitus
Circulatory diseases	Acute cardiac disease<br>Other and unspecified cardiac disease
Non-Communicable Diseases	Chronic obstructive pulmonary disease<br>Other and unspecified NCD
Renal diseases	Renal failure
Accidental	Accidental fall<br>Road traffic accident<br>Accidental expos to smoke fire & flame
Indeterminate	Cause of death unknown
Stroke	Stroke
